# Electrocardiographic changes with the onset of diabetes and the impact of aerobic exercise training in the Zucker Diabetic Fatty (ZDF) rat

**DOI:** 10.1186/1475-2840-9-56

**Published:** 2010-09-22

**Authors:** Lisa VanHoose, Youssef Sawers, Rajprasad Loganathan, James L Vacek, Lisa Stehno-Bittel, Lesya Novikova, Muhammed Al-Jarrah, Irina V Smirnova

**Affiliations:** 1 Department of Physical Therapy and Rehabilitation Science, University of Kansas Medical Center, MS 2002, 3901 Rainbow Blvd, Kansas City, KS 66160, USA; 2 Mid America Cardiology, University of Kansas Hospital, MS 4023, 3901 Rainbow Blvd, Kansas City, KS 66160, USA; 3 Department of Allied Medical Sciences, Faculty of Applied Medical Sciences, Jordan University of Science and Technology, Irbid 22110, Jordan

## Abstract

**Background:**

Early markers of diabetic autonomic neuropathy (DAN) in an electrocardiogram (ECG) include elevated R wave amplitudes, widening of QT_c _intervals and decreased heart rate variability (HRV). The severity of DAN has a direct relationship with mortality risk. Aerobic exercise training is a common recommendation for the delay and possible reversal of cardiac dysfunction. Limited research exists on ECG measures for the evaluation of aerobic exercise training in Zucker Diabetic Fatty (ZDF) rat, a model of type 2 diabetes. The objective of this study was to assess whether aerobic exercise training may attenuate diabetes induced ECG changes.

**Methods:**

Male ZDF (obese fa/fa) and control Zucker (lean fa/+) rats were assigned to 4 groups: sedentary control (SC), sedentary diabetic (SD), exercised control (EC) and exercised diabetic (ED). The exercised groups began 7 weeks of treadmill training after the development of diabetes in the ED group. Baseline (prior to the training) and termination measurements included body weight, heart weight, blood glucose and glycated hemoglobin levels and ECG parameters. One way repeated measures ANOVA (group) analyzed within and between subject differences and interactions. Pearson coefficients and descriptive statistics described variable relationships and animal characteristics.

**Results:**

Diabetes caused crucial changes in R wave amplitudes (p < 0.001), heart rate variability (p < 0.01), QT intervals (p < 0.001) and QT_c _intervals (p < 0.001). R wave amplitude augmentation in SD rats from baseline to termination was ameliorated by exercise, resulting in R wave amplitude changes in ED animals similar to control rats. Aerobic exercise training neither attenuated QT or QT_c _interval prolongation nor restored decreases in HRV in diabetic rats.

**Conclusion:**

This study revealed alterations in R wave amplitudes, HRV, QT and QT_c _intervals in ZDF rats. Of these changes, aerobic exercise training was able to correct R wave amplitude changes. In addition, exercise has beneficial effect in this diabetic rat model in regards to ECG correlates of left ventricular mass.

## Background

Cardiovascular disease (CVD) accounts for the majority of deaths for people with type 2 diabetes mellitus. CVD is a broad term which includes any condition causing pathological changes in blood vessels, cardiac muscle or valves and cardiac rhythm. The electrocardiogram (ECG) offers a quick, non-invasive clinical and research screen for the early detection of CVD.

Electrocardiographic changes in raw and corrected QT intervals and R wave amplitudes are early indicators of evolving CVD and increased cardiovascular risk. Prolonged QT and QT_c _intervals are considered reliable predictors of heart disease and fatal ventricular arrhythmias [1-3]. A positive linear relationship exists between QT_c _interval prolongation and diabetic cardiac autonomic neuropathy (DAN) severity in diabetic population [4]. Heart rate variability (HRV), one indicator of DAN, decreases with diabetes which indicates increased mortality risk [5]. QT and QT_c _interval abnormalities reflect changes in cardiac architecture. A positive correlation between QT or QT_c _interval prolongation and left ventricular (LV) mass has been reported [6-8]. LV hypertrophy presents as exaggerated R wave amplitudes on ECG recordings. Elevated R wave amplitudes are an independent risk factor for cardiovascular events [9]. LV hypertrophy and QT interval alterations coupled with decreased cardiac function are commonly observed with diabetes related CVD [10].

Non pharmacological interventions for CVD focus primarily on lifestyle changes with physical activity as the primary focus and a risk reduction strategy. Physical activity reduces QT_c _interval prolongation and cardiac dysfunction in healthy subjects [11,12]. Exercise lowers heart rate and increases HRV in healthy and diseased populations [13,14]. Physical activity can serve as potent prescription in the delay and attenuation of the CVD complications for persons with type 2 diabetes but additional comparative studies are needed regarding the cardiac response to exercise under diabetic conditions at various time points of the disease.

The Zucker Diabetic Fatty (ZDF) rat is a model of type 2 diabetes. The ZDF rat develops hyperglycemia and hyperlipidemia by week 8 and overt diabetes by week 12. The progression mimics the obesity-related insulin resistance and inflammation seen in humans [15,16]. The ZDF rat is commonly used to investigate prevention of diabetes; however, research related to the diabetic heart disease including ECG studies with this animal model is limited. We hypothesized that ECG changes occur in ZDF rats early in the disease process and aerobic exercise training is able to alleviate the changes. We detected changes in ECG parameters that were partially corrected by exercise training. Our findings add to the characterization of the ZDF model for studying type 2 diabetes effects on the heart and explore the benefits of an early exercise intervention in the presentation and progression of diabetes related CVD.

## Methods

### Animals

Male Zucker Diabetic Fatty (fa/fa) rats of 11 weeks were utilized for the study. Male lean, age-matched Zucker (fa/+) rats (both from Charles River Laboratory, Saint Louis, MO) served as non-diabetic controls. The animals were allowed food and water ad libitum and were placed on a 12:12 light-dark cycle. As per vendor's recommendations, the animals were fed Purina 5008 diet during the entire study for the development of a disease process resembling type 2 diabetes and its complications. All animal procedures were performed according to the University of Kansas Medical Center Institutional Animal Care and Use Committee guidelines and an approved Animal Care and Use Protocol.

### Measurements

Body weights and blood glucose levels were measured weekly on all animals. Blood glucose levels were measured from rat tail using Accu-Check Active meter (Roche Diagnostics, Indianapolis, IN). Glycated hemoglobin (HbA1c) levels were measured at the end of the experiment using antibody-based A1cNow meter (Metrika, Sunnyvale, CA). When rats had blood glucose or HbA1c levels higher than detectable by the method used, we used the highest detectable value (600 mmol/L or 13%, respectively) for statistical purposes. Animals were killed within 36 hours of the last exercise training episode.

### Aerobic exercise training

The rats started a treadmill exercise program at 12 weeks of age, immediately after the onset of diabetes, and continued exercising for 7 weeks. Four groups of rats were used: sedentary control (SC, n = 12), sedentary diabetic (SD, n = 10), exercised control (EC, n = 10) and exercised diabetic (ED, n = 12). This training protocol has been published by our group previously for a rat model of type 1 diabetes [17] and was adapted for the obese diabetic rats as they were unable to perform at that intensity level. During the first week of training the animals ran at 10 m/min with time increased from 10 min per day to 40 min per day at the end of the week. The progression allowed the rats to acclimate to the treadmill. Starting at week two and until the completion of the training session the rats ran at 15 m/min, for 40 minutes, 5 days per week. In order to accommodate for the disease progression in the diabetic rats, any animals showing signs of fatigue were allowed breaks of a few minutes until they were able to continue, for a total run time of 40 min per day. All rats assigned to the exercise groups completed the exercise training protocol.

### Electrocardiogram (ECG) assessment

Animals received ketamine (60 mg/kg) and xylazine (7 mg/kg) prior to the resting ECG recording. ECG leads I, II, III, aVR, aVL, aVF were recorded with surface electrodes (ADInstruments, Colorado Springs, CO). Measurements were collected at baseline, prior to training and after 7 weeks of exercise training. The mean value for each rat was obtained from four values consisting of four consecutive cardiac cycles using LabChart software (ADInstruments, Colorado Springs, CO). Corrected QT (QT_C_) was calculated with mean values and the Bazett's Formula (QT_c _= QT Interval/√ (RR interval) [18]. The heart rate (bpm) for each animal was calculated by dividing 60 by the mean RR interval. Heart rate variability (HRV) was measured as the standard deviation of the RR intervals.

### Statistical analysis

Descriptive statistics was performed on animals' means for each group. One way repeated measures ANOVA (group) analyzed within and between subject differences and interactions. Single time point measurements or change scores were completed with one way ANOVA (group) with Least Significant Difference (LSD) post-hoc analysis. Pearson correlations were utilized for relationship values. Partial eta squared values are reported for the proportion of total variability attributed to a factor. Statistics were conducted with PASW Version 17 software (SPSS Inc, Chicago, IL, USA). Significance was measured at p < 0.05. Results are presented as means ± standard errors. The effect size of baseline body weights was large with Cohen's d = 2.8 and power was greater than 90% with sample sizes of 10-12 per group.

## Results

### Animal characteristics

**Figure 1:**
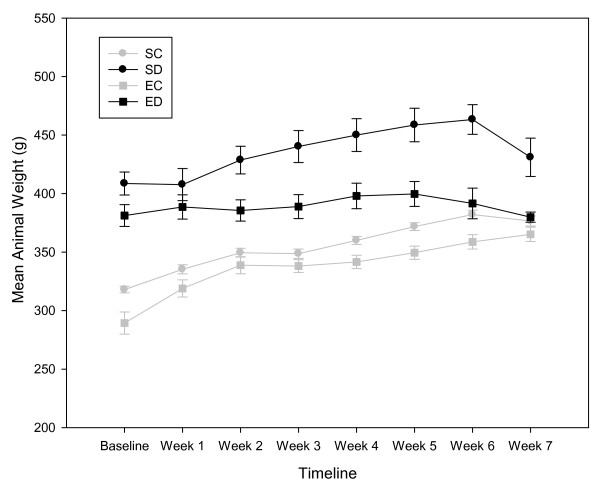
**Weekly mean body weight measurements**. Data are means ± SEs for each group with n = 12 for sedentary diabetic (SD) and exercised control (EC); and n = 10 for sedentary control (SC) and exercise diabetic (ED) rats.

A summary of animal characteristics are reported in Table 1 and weekly body weights are plotted in Figure 1. A significant difference in body weight between diabetic and control animals was observed at baseline and termination time points, *F*(3,40) = 19.37; *p *< 0.001. The mean difference between baseline and termination body weights indicated that control animals gained approximately 14% of their baseline body weights compared to a 5% gain for diabetic animals, *p *< 0.001. Diabetic animals outweighed their control counterparts by 9-12% at termination even with the discrepancy in weight gain. Although ED rats weighed less than SD animals at the termination, the ED rats were significantly heavier than SC and EC animals, *p *= 0.016 and *p *< 0.001 respectively. However, aging accounted for 64% of the variance in body weights as evident by average weight gains of 16-57 grams in all animal groups, *F*(1,39) = 71.29; *p *< 0.001. A moderate relationship between body weight and blood glucose levels existed at the baseline *r*(44) = 0.56; *p *< 0.001 and termination *r*(44) = 0.41; *p *< 0.01. The main effect of the group on blood glucose levels was modified by aging, *F*(1,39) = 3.02; *p *< 0.04. Although all animals had an increase in blood glucose levels, ED animals had only a 10% change compared to the 21% change of SD rats. HbA1C levels were elevated in diabetic groups compared to control groups, *F*(3,40) = 99.27; *p *< 0.001. Exercise did not attenuate HbA1C levels in ED animals, *p *= 0.92. Hyperglycemia is a factor in left ventricular hypertrophy development and progression but a correlation between A1C levels and heart weight/body weight ratios was not observed, *r*(44) = -0.17 *p *= 0.27. Heart weight/body weight ratios were similar for the animal groups, *F*(3,40) = 1.39; *p *= 0.26.

**Table 1:** Animal characteristics at baseline and termination

	Sedentary Control (SC) n = 10		Sedentary Diabetic (SD) n = 12		Exercised Control (EC) n = 12		Exercised Diabetic (ED) n = 10	
	
	Baseline	Term	Baseline	Term	Baseline	Term	Baseline	Term
**Body weight (g)**	326 ± 13	381 ± 13	409 ± 34^a, b, f^	457 ± 41^a, b, f^	309 ± 30^c, f^	366 ± 22^c, f^	374 ± 24^b, c, e^	390 ± 23^b, c^

**Blood glucose (mmol/L)**	115 ± 9	145 ± 9	433 ± 144^a, f^	579 ± 35^a, f^	111 ± 11^c, f^	156 ± 12^c, f^	491 ± 136^c^	584 ± 22^c, e^

**HbA1c (%)**	ND	4.2 ± 0.3	ND	13.0 ± 0.0^a, f^	ND	4.7 ± 0.3^c, d, f^	ND	13.0 ± 0.0^c, e^

**Heart rate (bpm)**	355 ± 148	416 ± 190	393 ± 68	423 ± 84	358 ± 139	408 ± 100	397 ± 73	413 ± 81

**Heart weight (g)**	1.24 ± 0.12		1.29 ± 0.09^f^		1.15 ± 0.08^d, f^		1.22 ± 0.10	

**Heart weight/Body weight (mg/g)**	3.27 ± 0.04		3.02 ± 0.03		3.16 ± 0.02		3.09 ± 0.03	

Data are means ± SEs for each group with n as indicated. Statistical significance: Between group differences at baseline or termination (term) of *p *≤ 0.05; ND - not determined; ^a^SC vs SD, ^b^SD vs ED, ^c^EC vs ED, ^d^SC vs EC, ^e^SC vs ED and ^f^SD vs EC.

### ECG wave amplitudes

#### R wave amplitude

**Figure 2:**
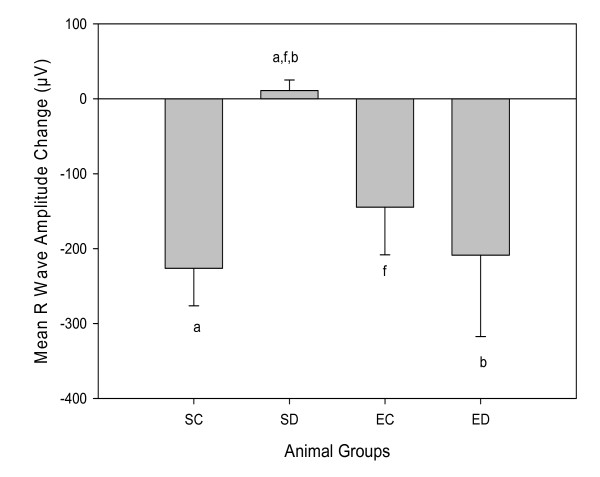
**Mean R wave amplitude changes**. Data are means ± SEs for each group with n = 12 for sedentary diabetic (SD) and exercised control (EC); and n = 10 for sedentary control (SC) and exercise diabetic (ED) rats. Statistical significance with *p *≤ 0.05; ^a^SC vs SD, ^b^SD vs ED and ^f^SD vs EC.

R wave amplitudes were similar for groups at baseline except for the SD groups, *F*(3,40) = 15.16, *p *< 0.001. Therefore, statistical analyses used the change value for R wave amplitude to account for the difference at baseline. Gains in R wave amplitudes from baseline to termination of the experiment were only observed in the SD animals as reported in Figure 2. The SD rats had a 17% increase in R wave amplitude, suggesting left ventricular hypertrophy. A reduction in R wave amplitude was found in ED animals at termination. ED rats had change values similar to SC and EC animals, *F*(3,40) = 4.13, *p *= 0.84 and *p *= 0.87.

#### T wave amplitude

**Figure 3:**
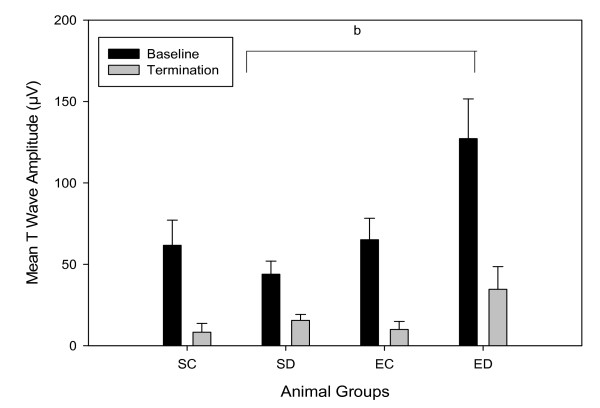
**Mean T wave amplitudes**. Data are means ± SEs for each group with n = 12 for sedentary diabetic (SD) and exercised control (EC); and n = 10 for sedentary control (SC) and exercise diabetic (ED) rats. Statistical significance with *p *≤ 0.05; ^b^SD vs ED.

Due to the significant differences between diabetic groups at baseline, we analyzed the change of T wave amplitudes from baseline to termination (Figure 3). T wave amplitudes changes were similar in the four groups, *F*(3,40) = 1.81, *p *= 0.16.

#### P wave amplitude

**Figure 4:**
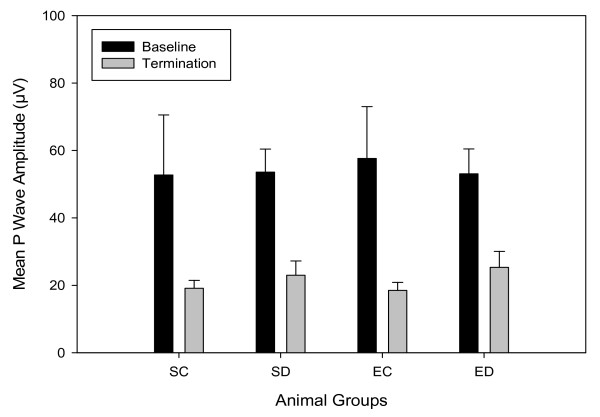
**Mean P wave amplitudes**. Data are means ± SEs for each group with n = 12 for sedentary diabetic (SD) and exercised control (EC); and n = 10 for sedentary control (SC) and exercise diabetic (ED) rats. Statistical significance (*p *< 0.001) between baseline and termination values was found in all four animal groups.

P wave amplitudes were similar between control and diabetic animals at baseline and termination, *F*(3,40) = 0.40, *p *= 0.99 (Figure 4). The amplitudes were decreased between baseline and termination in all four animal groups, as evident by time modifying the group effect, *F*(1,40) = 25.05, *p *< 0.001.

### ECG intervals

#### RR intervals

A significant shortening of RR intervals and increased heart rates was observed in SD group at baseline when compared to the SC group, *F*(1,38) = 8.83, p < 0.01 (Table 2). At termination, only a trend toward tachycardia was observed SD animals compared to SC animals, *F*(3,36) = 2.79, p = 0.06. Exercise did not reverse the heart rate pattern for diabetic animals, *p *= 0.92. Table 2 shows that all animal groups had various levels of RR interval shortening at termination, but statistically an aging effect was not observed, *F*(1,38) = 0.34, p = 0.56. Decreased HRV was observed in all diabetic animals, sedentary and exercised compared to control animals *F*(1,38) = 1662.23, *p *< 0.01. HRV was not improved with exercise, *p *= 0.91 as calculated with the termination RR intervals of the ED animals.

**Table 2:** ECG interval measurements

	Sedentary Control (SC) n = 10		Sedentary Diabetic (SD) n = 12		Exercised Control (EC) n = 12		Exercised Diabetic (ED) n = 10	
	
	Baseline	Term	Baseline	Term	Baseline	Term	Baseline	Term
**RR interval (sec)**	0.195 ± 0.077	0.152 ± 0.041	0.158 ± 0.033	0.132 ± 0.012	0.191 ± 0.073	0.158 ± 0.048	0.157 ± 0.036	0.135 ± 0.012

**QRS interval (sec)**	0.016 ± 0.005	0.022 ± 0.002	0.020 ± 0.002^a, f^	0.022 ± 0.004	0.016 ± 0.004^c, f^	0.023 ± 0.003	0.020 ± 0.002^c, e^	0.021 ± 0.004

**QT interval (sec)**	0.043 ± 0.012	0.040 ± 0.005	0.064 ± 0.013^a, f^	0.051 ± 0.012^a, f^	0.045 ± 0.012^c, f^	0.041 ± 0.006^c, f^	0.062 ± 0.011^c, e^	0.052 ± 0.013^c, e^

**QT_c _interval (sec)**	0.105 ± 0.043	0.105 ± 0.020	0.163 ± 0.033^a, f^	0.134 ± 0.034^a, f^	0.111 ± 0.043^c, f^	0.106 ± 0.018^c, f^	0.159 ± 0.026^c, e^	0.134 ± 0.037^c, e^

**PR interval (sec)**	0.032 ± 0.010	0.030 ± 0.006	0.045 ± 0.011^a, f^	0.034 ± 0.008	0.036 ± 0.012^c, f^	0.030 ± 0.005	0.046 ± 0.011^c, e^	0.034 ± 0.009

Data are means ± SEs for each group with n as indicated. Statistical significance: Between group differences at baseline or termination (term) of *p *≤ 0.05; ^a^SC vs SD, ^c^EC vs ED, ^e^SC vs ED and ^f^SD vs EC.

#### QRS intervals

Widening of QRS intervals, a sign of abnormal intraventricular conduction, was found at baseline in diabetic animals, *F*(3,40) = 3.72, *p *< 0.01 (Table 2). An 18% difference existed in the duration of QRS intervals between the SC and SD animals. At termination, QRS intervals increased 6-9% for SD animals in comparison to 27-29% increases in SC animals. These changes implied that aging interacted with the main effect of the group, *F*(1,41) = 16.78, *p *< 0.001 and accounted for 29% of the variability of QRS intervals at termination. Therefore, the difference between SC and SD animals at baseline was lost at termination, *F*(3,40) = 0.57, *p *= 0.64. An impact of exercise on QRS intervals was not observed in the EC and ED animals.

#### QT and QT_c _intervals

SD animals presented with QT intervals (a measure of ventricular repolarization) 31% wider than SC animals at baseline, *F*(3,40) = 9.37, *p *< 0.001 (Table 2). After 7 weeks of exercise, QT intervals in ED animals remained widened, *F*(3,40) = 14.13, *p *= 0.85. At termination, the difference between SC and SD animals decreased to 21%. Aging accounted for 20% of the variability in termination QT intervals, but the group effect of diabetes accounted for 51% of the variability. An interaction between aging and group factors was not significant, and QT intervals were not affected by exercise in control or diabetic animals, *p *= 0.91 and *p *= 0.61.

QT_c _intervals (QT intervals corrected with RR intervals) were analyzed for an evaluation independent of heart rate. SD animals displayed significant widening of QT_c _intervals compared to SC animals, *F*(1,42) = 10.58, *p *< 0.001 (Table 2). Exercise did not attenuate the widening of QT_c _intervals in ED animals, *p *= 0.66. Compared to QT intervals, aging accounted for 85% of the variability in QT_c _intervals and 43% was due to group effects at termination. The interaction between time and group was significant, *F*(3,40) = 4.54, *p *< 0.01.

#### PR intervals

Baseline measurements revealed significant prolongation of PR intervals in SD animals compared to SC animals, *F*(1,39) = 5.40, *p *< 0.01 (Table 2). At termination, the difference between groups was not observed, *p *= 0.34. Exercise did not impact PR intervals in EC and ED animals.

## Discussion

CVD risk is increased up to four-fold in people with diabetes compared with their nondiabetic counterparts [19]. Researchers are aggressively trying to identify early detection methods and explore the factors contributing to diabetes related heart disease. The ZDF rat model is routinely used to investigate physiological and molecular hypotheses regarding diabetes and its related complications. Reports are available indicating cardiac dysfunction in the ZDF rat established primarily with hemodynamic or echocardiographic measurements or through experiments on the isolated heart [20-23]. However, limited information exists about the ECG changes that occur in the ZDF rat and its response to physical activity, specifically to aerobic exercise that is commonly recommended for those at risk or diagnosed with diabetes related heart disease [24,25]. Our project aimed to address this gap in the current literature.

Although we did not measure autonomic function directly, ECG indicators of autonomic dysfunction were observed in the ZDF rats at baseline with HRV alterations, tachycardia and QT interval prolongation. Autonomic innervations control HRV and cardiac function through a delicate balance of sympathetic and parasympathetic responses. Diabetes stimulates the sympathetic nervous system initially but prolonged exposure to hyperglycemia and elevated catecholamine levels cause a decrease in adrenergic receptors [26]. As diabetes creates a sympathetic predominance, it also produces a corresponding parasympathetic imbalance by denervation of the vagus nerve. Subclinical symptoms of DAN, primarily decreased HRV, are believed to appear in humans within one year of type 2 diabetes diagnosis and clinical presentations may not emerge until years into the diagnosis [27]. Bergstrom et al identified symptoms of DAN in type 1 diabetic patients with durations of diabetes as low as two months [28]. In our animal study, diminished HRV was noted in ECG recordings as early as within one week of diabetes onset for the ZDF.

Clinical DAN symptoms include resting tachycardia, exercise intolerance and orthostatic hypotension and heart rate syndromes [29]. Tachycardia was observed in diabetic animals at baseline, within a week of diabetes onset. Tachycardia is also a common finding in humans with uncontrolled diabetes [30]. Similarly, our animals were not treated for their hyperglycemia as evident by blood glucose and HbA1C levels. The role of tachycardia is controversial because research indicates that it may be a diabetic complication or a causative factor of diabetes. Nagaya et al argue that elevated resting heart rates and systolic blood pressure increase the risk for type 2 diabetes [31]. Another work by the same group indicated that prolonged QT_c _intervals were also an independent risk factor for the development of diabetes [32]. Our analysis indicates that widening of QT_c _intervals was present with the onset of diabetes at twelve weeks of age in ZDF rats. Thus abnormalities of ventricular repolarization are present at an early stage of diabetes in this model. An earlier time study would be useful to verify the presence of tachycardia and QT_c _interval prolongation during a pre-diabetic state. Of interest, several ECG parameters in our study showed an impact of aging, in agreement with findings by others [33] indicating cardiac and renal changes in the ZDF rat model with aging, up to 36 months.

P wave amplitudes were unaffected with the onset of diabetes. Future studies need to investigate the relationship between the dispersion of the P wave and the onset of diabetes. Obesity is commonly linked to diabetes and is reported to increase the dispersion of the P wave [34]. PR intervals were widened in diabetic animals at baseline, but normalized at termination. PR intervals are commonly associated with atrial fibrillation [35,36]; however our ECG assessments of diabetic animals did not indicate atrial dysfunction. Diabetes and exercise appeared to have no effect on T wave amplitudes. T wave amplitudes were skewed at baseline and therefore change scores were used for analysis. However, the morphology of the T wave has shown to have prognostic value for CVD, not amplitude changes [37,38].

After seven weeks of aerobic exercise, ED animals showed R wave amplitudes comparable to control animals. Only SD animals had an increase in R wave amplitude. Hyperglycemia is associated with left ventricular hypertrophy in type 2 diabetes [39]. The increase in R wave amplitude may indicate a change in left ventricular mass. With diabetes, pathological hypertrophy results from myocardial damage and fibrosis [40-42]. Fibrosis affects the filling and contractility of the ventricles. Subsequently, cardiac dysfunction presents as decreased activity tolerance, ejection fraction, cardiac output and heart failure [43]. However, other factors can alter R wave amplitudes, including electrical axis deviations, altered electrodes position and differences in chest wall thickness. Ideally, the ECG should be correlated with an echocardiography. Unfortunately, we were unable to use echocardiography approach, thus we recognize this as a limitation of this study. In analyzing other indications of heart hypertrophy, we found no difference in the heart weight/body weight ratio in our study. Alternatively, the LV weight/tibial length ratio has been validated as an index of cardiac hypertrophy in mature rats [44]. Due to the fact that the onset of diabetes in the ZDF rat occurs early in their age, we were restricted to using relatively young animals that were in the latter stages of their growth phase. Thus, normalizing heart size to the changing tibial bone length during growth may not have provided an accurate estimation in our study. Darmellah et al reported that normalization of the heart weight per body weight or tibial length resulted in similar measurements of cardiac hypertrophy in Goto-Kakizaki animals, another rat model of type 2 diabetes [45].

Aerobic exercise did not impact the hyperglycemia. Exercise is postulated to improve glucose uptake and decrease lipid accumulation in persons with controlled diabetes but the protective mechanism of exercise is lost if hyperglycemia persist [46]. A comparative study of fenobirate and metformin validated the role of lipid oxidation in the development and progression of diabetes related heart disease, with fenobirate decreasing triglycerides content and fibrosis in diabetic myocardium [47]. The switch in myocardial substrate from glucose to fatty acids has been shown to result in systolic and diastolic dysfunction in the ZDF model [48]. Exercise training has also been suggested to improve microcirculation through enhanced endothelial function [49] through normalizing glycemic levels. However, the severity of diabetes will determine if the body can adapt to the demands of exercise or regional flow has already been compromised beyond recovery [50]. Microcirculatory disturbances or small vessel disease may lead to declines in myocardial blood flow which could influence ECG parameters [51].

A 31% difference in QT intervals was reported between SC and SD animals at baseline. We hypothesize that cardiac remodeling was already in the process in the SD rats when we were taking the baseline measures, prior to any training, due to animals already displaying hyperglycemia and obesity associated with the genotype, in contrast to the SC rat. QTc intervals did not respond to exercise, but the chronic tachycardia shortened the intervals as a compensatory effect. Commonly, exercise may cause a decrease in heart rate and increased ventricular relaxation which presents as longer QT intervals. A study investigating the effects of a seven month endurance training program in dogs revealed an increase in QT intervals [52]. An acute resistance exercise resulted in a similar effect on QT_c _intervals [53]. Since our obese, diabetic animals were showing early signs of autonomic disturbance, exercise tolerance was lowered and animals required frequent rest breaks during our training program. The mode and duration of exercise might not have been sufficient for QT_c _interval adaptation. In a future study we will investigate longer durations of exercise training to see whether it may return diabetic animals to normal heart rates or restore HRV. Pagkalos et al reported improvements in cardiac autonomic function with six months of aerobic exercise training [54]. Another alternative is the evaluation of HRV during post-exercise recovery. Training may not affect resting HRV, but the benefits may be evident with the post-exercise recovery. This conclusion is supported by a study investigating cardiac autonomic function in women with and without diabetes [55].

## Conclusions

In summary, our investigation proved that ECG alterations do occur with diabetes in the ZDF rat. The alterations include prolongation of the QT_c _interval and tachycardia which constitute important electrophysiologic alterations in this animal model of diabetes. These modifications coupled with high R wave amplitude illustrate the early cardiac anatomic and electrophysiologic alterations in this diabetic model. After seven weeks of exercise training, R wave amplitude changes in diabetes were similar to control animals from baseline to termination. However, aging may have an impact on several ECG parameters and the ZDF model showed changes in atrial and ventricular conduction possibly due to an interaction of aging and group effects. Future studies are needed to investigate ECG changes in the ZDF model before the onset of diabetes which will provide additional information about the use of QT_c _intervals and HRV in the early detection of DAN.

## Competing interests

The authors declare that they have no competing interests.

## Authors' contributions

LVH performed data analysis and interpretation and drafted the manuscript. YS performed data analysis and interpretation. RL handled the animals, completed the electrocardiograms and participated in data analysis. JV participated in data analysis and interpretation and revision of the manuscript draft. LSB participated in the design of the study and animal handling, contributed to the interpretation of results and revision of the manuscript draft. LN participated in exercise training, data collection and coordination of the study. MAJ participated with the exercise training of the animals and data collection. IVS conceived the study, participated in the design, oversaw all the work, handled animals, performed data collection and revised the manuscript draft. All authors approved of the final manuscript.

## References

[B1] Gorodeski EZ Ishwaran H Blackstone EH Lauer MS Quantitative electrocardiographic measures and long-term mortality in exercise test patients with clinically normal resting electrocardiograms Am Heart J 2009 158 1 61 70 10.1016/j.ahj.2009.04.015 19540393 PMC4213951

[B2] Cardoso CR Salles GF Deccache W QTc interval prolongation is a predictor of future strokes in patients with type 2 diabetes mellitus Stroke 2003 34 9 2187 2194 10.1161/01.STR.0000085084.15144.66 12893949

[B3] Christensen PK Gall MA Major-Pedersen A Sato A Rossing P Breum L Pietersen A Kastrup J Parving HH QTc interval length and QT dispersion as predictors of mortality in patients with non-insulin-dependent diabetes Scand J Clin Lab Invest 2000 60 4 323 332 10.1080/003655100750046486 10943602

[B4] Mathur C Gupta D QTc prolongation in diabetes mellitus-An indicator of cardiac autonomic neuropathy Journal, Indian Academy Clinical Medicine 2006 7 2 130 132

[B5] Schroeder EB Chambless LE Liao D Prineas RJ Evans GW Rosamond WD Heiss G Diabetes, glucose, insulin, and heart rate variability: the Atherosclerosis Risk in Communities (ARIC) study Diabetes Care 2005 28 3 668 674 10.2337/diacare.28.3.668 15735206

[B6] Oikarinen L Nieminen MS Viitasalo M Toivonen L Wachtell K Papademetriou V Jern S Dahlof B Devereux RB Okin PM Relation of QT interval and QT dispersion to echocardiographic left ventricular hypertrophy and geometric pattern in hypertensive patients. The LIFE study. The Losartan Intervention For Endpoint Reduction J Hypertens 2001 19 10 1883 1891 10.1097/00004872-200110000-00025 11593111

[B7] Davey PP Barlow C Hart G Prolongation of the QT interval in heart failure occurs at low but not at high heart rates Clin Sci (Lond) 2000 98 5 603 610 10.1042/CS19990261 10781393

[B8] Pshenichnikov I Shipilova T Kaik J Volozh O Abina J Lass J Karai D QT dispersion in relation to left ventricular geometry and hypertension in a population study Scand Cardiovasc J 2003 37 2 87 90 12775307 10.1080/14017430310001708a

[B9] Nakamura K Okamura T Hayakawa T Kadowaki T Kita Y Okayama A Ueshima H Electrocardiogram screening for left high R-wave predicts cardiovascular death in a Japanese community-based population: NIPPON DATA90 Hypertens Res 2006 29 5 353 360 10.1291/hypres.29.353 16832156

[B10] Zhang X Wang X Li L Zhang G Gao Y Cui J An analysis of factors influencing electrocardiogram stress test for detecting coronary heart disease Chin Med J (Engl) 1999 112 7 590 592 11601249

[B11] Perhonen MA Haapalahti P Kivisto S Hekkala AM Vaananen H Swan H Toivonen L Effect of physical training on ventricular repolarization in type 1 long QT syndrome: a pilot study in asymptomatic carriers of the G589 D KCNQ1 mutation Europace 2006 8 10 894 898 10.1093/europace/eul083 16882680

[B12] Genovesi S Zaccaria D Rossi E Valsecchi MG Stella A Stramba-Badiale M Effects of exercise training on heart rate and QT interval in healthy young individuals: are there gender differences? Europace 2007 9 1 55 60 10.1093/europace/eul145 17224424

[B13] Tuomainen P Peuhkurinen K Kettunen R Rauramaa R Regular physical exercise, heart rate variability and turbulence in a 6-year randomized controlled trial in middle-aged men: the DNASCO study Life Sci 2005 77 21 2723 2734 10.1016/j.lfs.2005.05.023 15978638

[B14] Sandercock GR Bromley PD Brodie DA Effects of exercise on heart rate variability: inferences from meta-analysis Med Sci Sports Exerc 2005 37 3 433 439 10.1249/01.MSS.0000155388.39002.9D 15741842

[B15] Schmidt RE Dorsey DA Beaudet LN Peterson RG Analysis of the Zucker Diabetic Fatty (ZDF) type 2 diabetic rat model suggests a neurotrophic role for insulin/IGF-I in diabetic autonomic neuropathy Am J Pathol 2003 163 1 21 28 12819007 10.1016/S0002-9440(10)63626-7 PMC1868158

[B16] Leonard BL Watson RN Loomes KM Phillips AR Cooper GJ Insulin resistance in the Zucker diabetic fatty rat: a metabolic characterisation of obese and lean phenotypes Acta Diabetol 2005 42 4 162 170 10.1007/s00592-005-0197-8 16382303

[B17] Searls YM Smirnova IV Fegley BR Stehno-Bittel L Exercise attenuates diabetes-induced ultrastructural changes in rat cardiac tissue Med Sci Sports Exerc 2004 36 11 1863 1870 10.1249/01.MSS.0000145461.38224.EC 15514499

[B18] Heffernan KS Jae SY Fernhall B Heart rate recovery after exercise is associated with resting QTc interval in young men Clin Auton Res 2007 17 6 356 363 10.1007/s10286-007-0450-z 18049832

[B19] Preis SR Hwang SJ Coady S Pencina MJ D'Agostino RB Sr Savage PJ Levy D Fox CS Trends in all-cause and cardiovascular disease mortality among women and men with and without diabetes mellitus in the Framingham Heart Study, 1950 to 2005 Circulation 2009 119 13 1728 1735 10.1161/CIRCULATIONAHA.108.829176 19307472 PMC2789419

[B20] Boudina S Abel ED Diabetic cardiomyopathy revisited Circulation 2007 115 25 3213 3223 10.1161/CIRCULATIONAHA.106.679597 17592090

[B21] Baynes JW Murray DB The metal chelators, trientine and citrate, inhibit the development of cardiac pathology in the Zucker diabetic rat Exp Diabetes Res 2009 2009 696378 19390595 10.1155/2009/696378 PMC2669293

[B22] Radovits T Korkmaz S Loganathan S Barnucz E Bomicke T Arif R Karck M Szabo G Comparative investigation of the left ventricular pressure-volume relationship in rat models of type 1 and type 2 diabetes mellitus Am J Physiol Heart Circ Physiol 2009 297 1 H125 133 10.1152/ajpheart.00165.2009 19429826

[B23] van den Brom CE Bosmans JW Vlasblom R Handoko LM Huisman MC Lubberink M Molthoff CF Lammertsma AA Ouwens MD Diamant M Diabetic cardiomyopathy in Zucker diabetic fatty rats: the forgotten right ventricle Cardiovasc Diabetol 2010 9 25 10.1186/1475-2840-9-25 20550678 PMC2898761

[B24] Mizuno R Fujimoto S Saito Y Nakamura S Exercise-induced delayed onset of left ventricular early relaxation in association with coronary microcirculatory dysfunction in patients with diabetes mellitus J Card Fail 2010 16 3 211 217 10.1016/j.cardfail.2009.10.024 20206895

[B25] Chipkin SR Klugh SA Chasan-Taber L Exercise and diabetes Cardiol Clin 2001 19 3 489 505 10.1016/S0733-8651(05)70231-9 11570119

[B26] Scott LA Kench PL Cardiac autonomic neuropathy in the diabetic patient: does 123I-MIBG imaging have a role to play in early diagnosis? J Nucl Med Technol 2004 32 2 66 71 15175402

[B27] Vinik AI Maser RE Mitchell BD Freeman R Diabetic autonomic neuropathy Diabetes Care 2003 26 5 1553 1579 10.2337/diacare.26.5.1553 12716821

[B28] Bergstrom B Lilja B Osterlin S Sundkvist Autonomic neuropathy in type 1 diabetes: influence of diabetes and other complications Acta Med Scand 2009 222 2 147 154 10.1111/j.0954-6820.1987.tb10652.x 3673667

[B29] Vinik AI Ziegler D Diabetic cardiovascular autonomic neuropathy Circulation 2007 115 3 387 397 10.1161/CIRCULATIONAHA.106.634949 17242296

[B30] Kitabchi AE Umpierrez GE Murphy MB Kreisberg RA Hyperglycemic crises in adult patients with diabetes: a consensus statement from the American Diabetes Association Diabetes Care 2006 29 12 2739 2748 10.2337/dc06-9916 17130218

[B31] Nagaya T Yoshida H Takahashi H Kawai M Resting heart rate and blood pressure, independent of each other, proportionally raise the risk for type-2 diabetes mellitus Int J Epidemiol 2010 39 1 215 222 10.1093/ije/dyp229 19564246

[B32] Nagaya T Yoshida H Takahashi H Kawai M Heart rate-corrected QT interval in resting ECG predicts the risk for development of type-2 diabetes mellitus Eur J Epidemiol 2010 25 3 195 202 10.1007/s10654-009-9423-y 20066475

[B33] Baynes J Murray DB Cardiac and renal function are progressively impaired with aging in Zucker diabetic fatty type II diabetic rats Oxid Med Cell Longev 2009 2 5 328 334 10.4161/oxim.2.5.9831 20716921 PMC2835922

[B34] Seyfeli E Duru M Kuvandik G Kaya H Yalcin F Effect of obesity on P-wave dispersion and QT dispersion in women Int J Obes (Lond) 2006 30 6 957 961 10.1038/sj.ijo.0803233 16432544

[B35] Homoud MK ACP Journal Club: Prolonged PR intervals were associated with increased risk for atrial fibrillation, pacemaker implantation, and mortality Ann Intern Med 2009 151 10 JC5 13 http://www.annals.org/content/151/10/JC5-13.long 19920258 10.7326/0003-4819-151-10-200911170-02013

[B36] Lorsheyd A de Lange DW Hijmering ML Cramer MJ van de Wiel A PR and OTc interval prolongation on the electrocardiogram after binge drinking in healthy individuals Neth J Med 2005 63 2 59 63 15766009

[B37] Nair D Tan SY Gan HW Lim SF Tan J Zhu M Gao H Chua NH Peh WL Mak KH The use of ambulatory tonometric radial arterial wave capture to measure ambulatory blood pressure: the validation of a novel wrist-bound device in adults J Hum Hypertens 2008 22 3 220 222 10.1038/sj.jhh.1002306 17992251

[B38] Huang HC Lin LY Yu HY Ho YL Risk stratification by T-wave morphology for cardiovascular mortality in patients with systolic heart failure Europace 2009 11 11 1522 1528 10.1093/europace/eup294 19819880

[B39] Goraksha-Hicks P Rathmell JC TGF-beta: a new role for an old AktTOR Dev Cell 2009 17 1 6 8 10.1016/j.devcel.2009.07.004 19619487 PMC2789270

[B40] Fukui S Fukumoto Y Suzuki J Saji K Nawata J Shinozaki T Kagaya Y Watanabe J Shimokawa H Diabetes mellitus accelerates left ventricular diastolic dysfunction through activation of the renin-angiotensin system in hypertensive rats Hypertens Res 2009 32 6 472 480 10.1038/hr.2009.43 19390543

[B41] Kannel WB Prevalence and natural history of electrocardiographic left ventricular hypertrophy Am J Med 1983 75 3A 4 11 10.1016/0002-9343(83)90111-0 6226193

[B42] Seferovic PM Lalic NM Seferovic JP Jotic A Lalic K Ristic AD Simeunovic D Radovanovic G Vujisic-Tesic B Ostajic MU [Diabetic cardiomyopathy: old disease or new entity?] Srp Arh Celok Lek 2007 135 9-10 576 582 18088046

[B43] Kannel WB Levy D Cupples LA Left ventricular hypertrophy and risk of cardiac failure: insights from the Framingham Study J Cardiovasc Pharmacol 1987 10 Suppl 6 S135 140 2485019

[B44] Yin FC Spurgeon HA Rakusan K Weisfeldt ML Lakatta EG Use of tibial length to quantify cardiac hypertrophy: application in the aging rat Am J Physiol 1982 243 6 H941 947 6216817 10.1152/ajpheart.1982.243.6.H941

[B45] Darmellah A Baetz D Prunier F Tamareille S Rucker-Martin C Feuvray D Enhanced activity of the myocardial Na+/H+ exchanger contributes to left ventricular hypertrophy in the Goto-Kakizaki rat model of type 2 diabetes: critical role of Akt Diabetologia 2007 50 6 1335 1344 10.1007/s00125-007-0628-x 17429605

[B46] Sato Y Diabetes and life-styles: role of physical exercise for primary prevention Br J Nutr 2000 84 Suppl 2 S187 190 11242467 10.1079/096582197388662

[B47] Forcheron F Basset A Abdallah P Del Carmine P Gadot N Beylot M Diabetic cardiomyopathy: effects of fenofibrate and metformin in an experimental model--the Zucker diabetic rat Cardiovasc Diabetol 2009 8 16 10.1186/1475-2840-8-16 19317897 PMC2664796

[B48] van den Brom CE Huisman MC Vlasblom R Boontje NM Duijst S Lubberink M Molthoff CF Lammertsma AA van der Velden J Boer C Altered myocardial substrate metabolism is associated with myocardial dysfunction in early diabetic cardiomyopathy in rats: studies using positron emission tomography Cardiovasc Diabetol 2009 8 39 10.1186/1475-2840-8-39 19624828 PMC2722582

[B49] Erbs S Hollriegel R Linke A Beck EB Adams V Gielen S Mobius-Winkler S Sandri M Krankel N Hambrecht R Exercise training in patients with advanced chronic heart failure (NYHA IIIb) promotes restoration of peripheral vasomotor function, induction of endogenous regeneration, and improvement of left ventricular function Circ Heart Fail 2010 3 4 486 494 10.1161/CIRCHEARTFAILURE.109.868992 20430934

[B50] Joshi D Shiwalkar A Cross MR Sharma SK Vachhani A Dutt C Continuous, non-invasive measurement of the haemodynamic response to submaximal exercise in patients with diabetes mellitus: evidence of impaired cardiac reserve and peripheral vascular response Heart 2010 96 1 36 41 10.1136/hrt.2009.177113 19850585 PMC3272706

[B51] Cosyns B Droogmans S Hernot S Degaillier C Garbar C Weytjens C Roosens B Schoors D Lahoutte T Franken PR Effect of streptozotocin-induced diabetes on myocardial blood flow reserve assessed by myocardial contrast echocardiography in rats Cardiovasc Diabetol 2008 7 26 10.1186/1475-2840-7-26 18764943 PMC2546381

[B52] Constable PD Hinchcliff KW Olson JL Stepien RL Effects of endurance training on standard and signal-averaged electrocardiograms of sled dogs Am J Vet Res 2000 61 5 582 588 10.2460/ajvr.2000.61.582 10803657

[B53] Heffernan KS Sosnoff JJ Jae SY Gates GJ Fernhall B Acute resistance exercise reduces heart rate complexity and increases QTc interval Int J Sports Med 2008 29 4 289 293 10.1055/s-2007-965363 17990212

[B54] Pagkalos M Koutlianos N Kouidi E Pagkalos E Mandroukas K Deligiannis A Heart rate variability modifications following exercise training in type 2 diabetic patients with definite cardiac autonomic neuropathy Br J Sports Med 2008 42 1 47 54 10.1136/bjsm.2007.035303 17526623

[B55] Figueroa A Baynard T Fernhall B Carhart R Kanaley JA Endurance training improves post-exercise cardiac autonomic modulation in obese women with and without type 2 diabetes Eur J Appl Physiol 2007 100 4 437 444 10.1007/s00421-007-0446-3 17406886

